# Longitudinal reciprocal associations between physical exercise and aggressive behavior in adolescents: a cross-lagged analysis

**DOI:** 10.3389/fpubh.2026.1898226

**Published:** 2026-07-21

**Authors:** Guanyun Zhong, Xiafang Li, Ting Zhang, Xinqi He, Delu Meng

**Affiliations:** Physical Education College, Jiangxi Normal University, Nanchang, China

**Keywords:** adolescents, aggressive behavior, cross-lagged analysis, multi-group analysis, physical exercise

## Abstract

**Objective:**

To examine the longitudinal reciprocal association between physical exercise and aggressive behavior among adolescents using a cross-lagged research design.

**Methods:**

A longitudinal study was conducted over 1 year at three time points (T1, T2, T3) involving 1,215 junior and senior high school students, using the Physical Activity Level Scale and the Aggression Behavior Scale.

**Results:**

Physical exercise among adolescents showed significant gender differences (*p* < 0.001), and gender differences in aggressive behavior were also significant (*p* < 0.05). Physical exercise and aggressive behavior exhibited stable, negative correlations across time (at T1, r = −0.183, *p* < 0.001; at T2, r = −0.189, *p* < 0.001; at T3, r = −0.223, *p* < 0.001). Cross-lagged analysis revealed a reciprocal longitudinal pattern: T1 physical exercise was negatively associated with T2 aggressive behavior (β = −0.097, *p* < 0.001), and T2 physical exercise was negatively associated with T3 aggressive behavior (β = −0.085, *p* < 0.01). Similarly, T1 aggressive behavior was negatively associated with T2 physical exercise (β = −0.053, *p* < 0.05), and T2 aggressive behavior was negatively associated with T3 physical exercise (β = −0.062, *p* < 0.05). These cross-lagged associations were consistent across genders and educational stages.

**Conclusion:**

This study reveals stable gender differences in both physical exercise and aggressive behavior among adolescents, and a bidirectional longitudinal association between the two, rather than a unidirectional one. Specifically, higher levels of physical exercise are associated with lower levels of aggressive behavior, and higher levels of aggressive behavior are associated with lower levels of physical exercise participation over time. Universal intervention strategies that address the reciprocal association between physical exercise and aggressive behavior may serve as a reference for adolescents across different genders and developmental stages.

## 1 Introduction

Aggressive behavior refers to intentional acts that cause physical or psychological harm to others (1). It is closely related to an individual's physical and mental health and serves as a factor influencing an individual's capacity for social development. Aggressive behavior is closely linked to an individual's emotional regulation abilities (2), social skills (3), and self-control (4). The World Health Organization's Preventing Youth Violence: Facts and Figures (5) and the Law of the People's Republic of China on the Protection of Minors (Revised in 2020) (6) explicitly state that preventing violence against children and adolescents is a critical public health issue, requiring families, schools, and society to jointly assume responsibility for protecting minors from violence. However, contemporary adolescents are prone to engaging in antisocial behavior (7). At the same time, due to inappropriate parenting practices, family dysfunction, and strained social relationships within contemporary families of origin, adolescents often lack emotional regulation and self-control, making them highly prone to conflicts and aggressive behavior when interacting with peers (8). The emergence of aggressive behavior can lead to social difficulties and mental health issues, disrupting normal daily life and triggering depression, anxiety, self-harm, and other problematic behaviors, which severely impact adolescents' physical and mental wellbeing (9). Adolescents in junior and senior high school are in a sensitive period of physical, mental, and social cognitive development. Preventing and reducing aggressive behavior among adolescents during this stage is not only crucial for their psychological wellbeing and social adaptation but also a necessary prerequisite for fostering citizens with a sense of social responsibility and a healthy personality.

The physical and mental health of adolescents has long been a key focus in the fields of public health and education. For many years, scholars have generally agreed that physical exercise is one of the most effective interventions for promoting adolescents' physical and mental health and social adaptation (10). Empirical research indicates that physical exercise helps develop general self-efficacy and emotional regulation strategies (11), promotes social connectedness (12), and enhances self-control while reducing aggressive behavior among adolescents (13). Generally speaking, adolescents who actively participate in physical exercise demonstrate better self-control (14), develop emotional stability and behavioral control (10), and exhibit fewer externalizing problem behaviors (such as impulsivity, hostility, and aggression) (15). In contrast, adolescents who reject, resist, or abandon participation in physical exercise are more prone to adverse psychological conditions such as anxiety and depression, exhibit rule-breaking and aggressive behaviors (16), and may even gradually develop behavioral disorders, leading to actual bullying and violent acts that trigger further conflicts (17), thereby provoking more aggressive behavior. Research indicates that adolescents who do not engage in physical exercise face a significantly increased risk of developing mood disorders, delusional thoughts, and visual perception disorders in adulthood (18). Negative behaviors often lead to negative psychological states and emotional experiences, thereby triggering interpersonal conflicts and social adaptation issues (19), increasing the likelihood of aggressive behavior. Based on this, it is inferred that physical exercise is an important associated factor for reducing aggressive behavior in adolescents.

However, scholars exploring the psychological mechanisms of adolescent aggressive behavior have reached divergent conclusions, suggesting that insufficient emotional regulation, poor social skills, and low levels of self-control are prerequisites for problem behavior (2–4), indicating that aggressive behavior may reflect a state of depleted psychological resources. Evidence indicates that adolescents with higher levels of aggressive behavior are more likely to experience loneliness and difficulties in school adjustment (20), and loneliness has been confirmed to be significantly negatively associated with physical activity levels in adolescents (21, 22). At the social level, adolescents with poor social skills are prone to interpersonal conflicts and often exhibit inappropriate behaviors such as defensive or aggressive language when interacting with others (3). Meanwhile, Zhou and Yang (23) demonstrated that adolescents with higher levels of social anxiety tend to have lower levels of physical activity participation (23). At the emotion regulation level, adolescents with poor emotion regulation skills are more prone to aggressive tendencies and often exhibit passive-aggressive behavior (2); when participating in group activities, they tend to display negative behaviors such as avoidance, apathy, withdrawal, covert truancy, and refusal to participate (24). At the self-control level, adolescents with poor self-control often display impulsive behaviors and struggle to regulate their emotions and actions when facing challenges such as setbacks, academic pressure, or interpersonal conflicts (4), and self-control has been confirmed to be significantly positively associated with physical activity participation, such that lower self-control significantly reduces engagement in physical activity (25). Aggressive behavior has been linked to deficiencies in emotional regulation, social skills, and self-control (2–4). Without effective intervention and guidance, aggressive behavior can severely inhibit and hinder an individual's ability to engage in activities. Based on this, it is hypothesized that aggressive behavior influences adolescents' actual participation in physical exercise.

**Figure 1:**
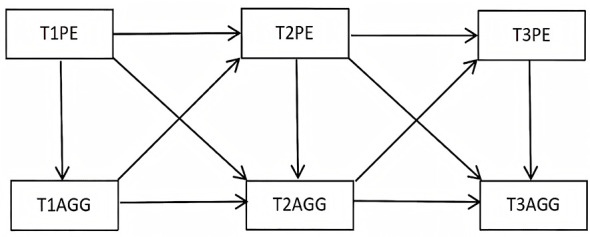
Hypothetical model. PE, physical exercise; AGG, aggressive behavior.

For adolescents in junior and senior high school, does physical exercise reduce aggressive behavior, or does aggressive behavior influence participation in physical exercise? A review of the existing literature reveals that the vast majority of studies have employed cross-sectional designs. Although recent cross-sectional studies have expanded their methodological approaches, for example, by using chain mediation models to examine the mediating roles of psychological capital and self-control (26), as well as life satisfaction, meaning in life, and depression (27) in the relationship between physical exercise and aggressive behavior, or by employing latent profile analysis to identify differences in aggressive behavior across subgroups with distinct emotional processing patterns or activity levels (28, 29), these cross-sectional designs remain inherently limited to revealing correlational relationships among variables and cannot establish temporal precedence. A small number of longitudinal studies have begun to address this question. For instance, Xu et al. (30) found that physical exercise negatively predicted subsequent aggressive behavior (30). Yue et al. (4) further demonstrated, through a three-wave longitudinal mediation model, that physical exercise indirectly predicted aggressive behavior via the mediating role of self-control (4). Wu et al. (31) found that leisure-time physical activity predicted lower levels of cyberbullying 1 year later through the mediating mechanism of peer relationships (31). However, these longitudinal studies share a common feature: they focus on mediating mechanisms, examining “through which pathways” physical exercise influences aggressive behavior, rather than directly testing whether a pure longitudinal association exists between the two variables, let alone addressing whether aggressive behavior conversely shows predictive effects on subsequent participation in physical exercise. This implies that the fundamental question of “which predicts which” remains inadequately answered. At the same time, adolescents in this developmental stage are becoming increasingly sensitive to gender, which may manifest as gender differences in behavioral patterns and social interactions. Therefore, whether gender differences also exist in adolescents' physical exercise and aggressive behavior is another question that requires clarification. It is well established that cross-lagged designs are effective in transcending the constraints of existing theories and testing longitudinal predictive relationships among variables. Accordingly, the present study begins by examining gender differences in adolescents' physical exercise and aggressive behavior and employs a three-wave cross-lagged design over 1 year to simultaneously test the bidirectional longitudinal associations between physical exercise and aggressive behavior (Figure 1), as well as to examine whether these associations are consistent across gender and educational stages (junior vs. senior high school), thereby addressing the research gaps identified above.

## 2 Materials and methods

### 2.1 Participants

**Figure 2:**
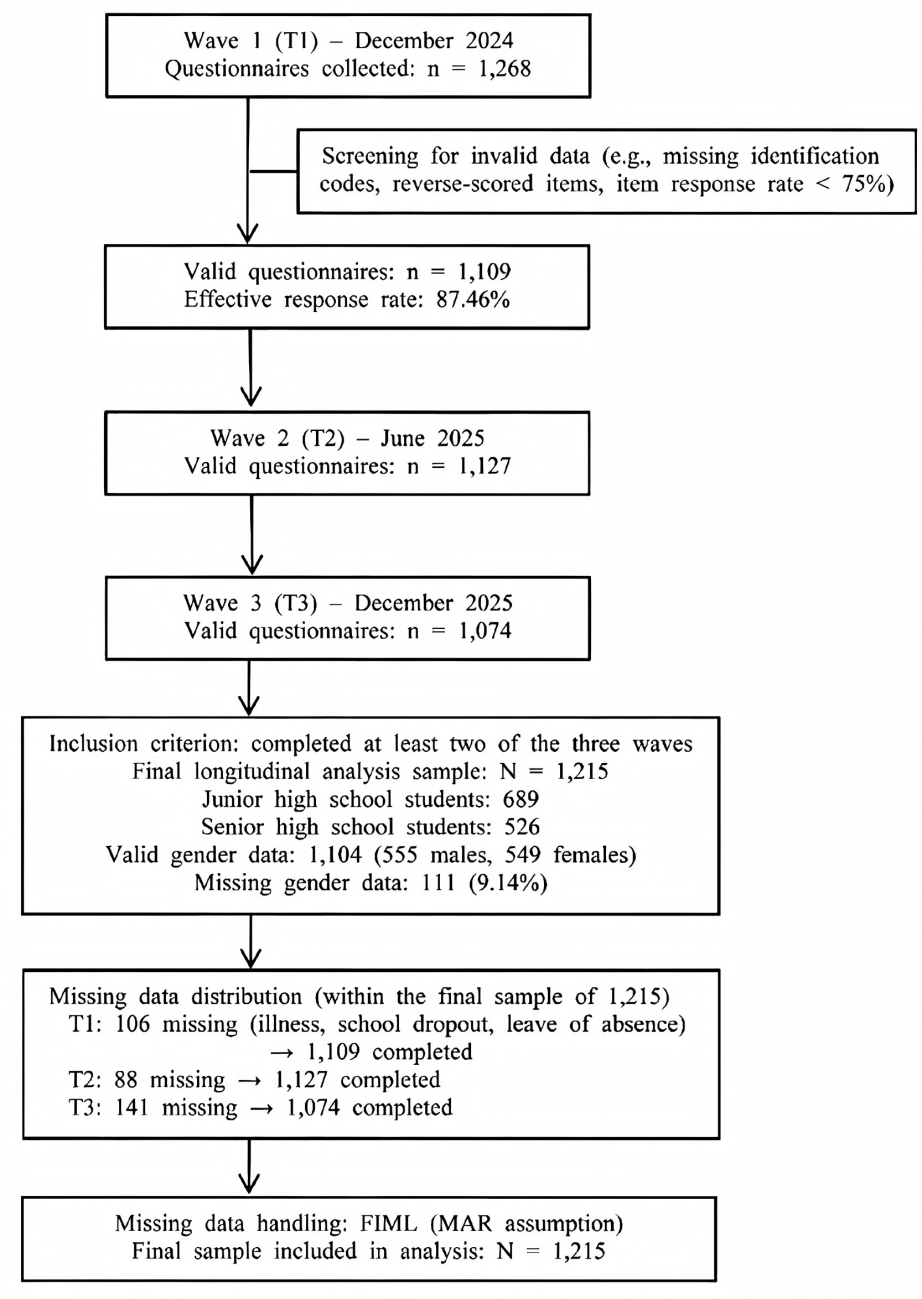
Flow diagram of participant recruitment and retention across the three-wave longitudinal study. FIML, full information maximum likelihood; MAR, missing at random.

Following the World Health Organization's (2024) definition of adolescents as individuals aged 10 to 19 (32), the study population consisted of students in junior and senior high schools; adhering to the principle of convenience sampling, eight schools (four junior high and four senior high) in Jiangxi Province were selected, and a longitudinal tracking study design was employed to survey participants three times over the course of 1 year. At T1 (December 2024), 1,268 questionnaires were collected; after screening for invalid data (e.g., missing identification codes, reverse-scored items, and item response rates below 75%), 1,109 valid questionnaires were retained, with an effective response rate of 87.46%, comprising 555 males, 549 females, and 5 participants with missing gender data. At T2 (June 2025), 1,127 valid questionnaires were retained, and at T3 (December 2025), 1,074 valid questionnaires were retained. Participants who completed at least two of the three assessments constituted the final longitudinal analysis sample (*N* = 1,215), consisting of 689 junior high school students and 526 senior high school students; among them, 1,104 participants had valid gender data (555 males and 549 females), while 111 participants had missing gender information. Within this final sample, 106 participants were absent at T1 (due to illness, school dropout, or leave of absence), 88 were absent at T2, and 141 were absent at T3. To handle missing data (including missing gender information), Full Information Maximum Likelihood (FIML) estimation was employed in Mplus 8.3 under the missing-at-random (MAR) assumption, using all available data to estimate model parameters. The detailed participant recruitment and retention process is presented in Figure 2. Prior to the survey, all participants were fully informed of the study's content and purpose, and informed consent was obtained from both the participants themselves and their parents or legal guardians, in compliance with research ethics guidelines.

### 2.2 Measures

#### 2.2.1 Physical exercise rating scale

This study adopted the Physical Activity Rating Scale (PARS-3) developed by Liang (33), which assesses the volume of physical exercise based on three dimensions—intensity, frequency, and duration—with the total score calculated as intensity × (duration – 1) × frequency, each dimension scored on a scale of 1 to 5, and with classification criteria of low exercise volume ≤ 19 points, moderate exercise volume 20–42 points, and high exercise volume ≥43 points; the test-retest reliability of this scale is 0.82 (33). In the current sample, the internal consistency coefficients (Cronbach's alpha) of the scale were 0.478 at T1, 0.594 at T2, and 0.559 at T3. Test-retest reliability was assessed using the intraclass correlation coefficient (ICC; two-way random effects, absolute agreement, single measures), with ICC = 0.738 between T1 and T2 and ICC = 0.732 between T2 and T3, indicating acceptable to good temporal stability across the three waves. Given that the PARS-3 total score is derived from a multiplicative formula combining intensity, duration, and frequency—dimensions that capture distinct aspects of physical activity rather than a single homogeneous construct—the relatively low alpha coefficients are within the expected range. Moreover, the significant negative correlations between physical exercise and aggressive behavior (T1: r = −0.183, *p* < 0.001; T2: r = −0.189, *p* < 0.001; T3: r = −0.223, *p* < 0.001) provide support for the criterion-related validity of the scale in the current sample.

#### 2.2.2 Aggression behavior scale

This study adopted the Brief Buss-Perry Aggression Questionnaire (BPAQ), developed by Webster et al. (57), based on the original 29-item version, which was subsequently localized for Chinese adolescents (34). The scale consists of 12 items across four dimensions: Physical Aggression (e.g., “If I have to use force to protect my rights, I will do so”), Verbal Aggression (e.g., “When I disagree with my friends, I tell them openly”), Anger (e.g., “I have trouble controlling my temper”), and Hostility (e.g., “When people are really nice to me, I wonder what they really want”), rated on a 5-point Likert scale (with Item 4 reverse-scored), with higher total scores indicating greater levels of aggressive behavior. In the present study, the internal consistency coefficients (Cronbach's alpha) were 0.645, 0.656, and 0.698 across the three waves. Confirmatory factor analysis (CFA) revealed that Item 11 (“My friends say that I am somewhat argumentative”) consistently exhibited factor loadings below 0.1 on the Verbal Aggression dimension across all three waves. Notably, similar findings have been reported in previous research: Liu et al. (35) found that all items except Item 11 had adequate factor loadings ranging from 0.51 to 0.75 (35); Zimonyi et al. (36) further pointed out that the Verbal Aggression factor of the BPAQ is difficult to define clearly, which may be attributed to the conceptual inaccuracy of the original items (36). Based on the above literature and content analysis, we argue that this item reflects emotional dysregulation in interpersonal conflict situations (being “argumentative”) rather than deliberate verbal attack, and thus is conceptually more aligned with the Anger dimension. Accordingly, Item 11 was reclassified under the Anger dimension. Following this adjustment, the Verbal Aggression dimension retained two items (Items 3 and 6), while the Anger dimension comprised four items (Items 4, 7, 11, and 12). To ensure model identification, the factor loadings of the two items in the Verbal Aggression dimension were fixed at 1 (37). Using robust maximum likelihood estimation (MLR), the three-wave CFA results indicated that the revised four-factor model demonstrated acceptable fit: at T1, CFI = 0.912, TLI = 0.881, RMSEA = 0.051, SRMR = 0.042; although TLI was slightly below 0.90 across waves, CFI, RMSEA, and SRMR all met the acceptable criteria, with all factor loadings significant (*p* < 0.001), supporting the construct validity of the revised model.

### 2.3 Data analysis

Valid samples were imported into SPSS 27.0, and reliability and validity tests were conducted using reliability analysis, correlation analysis, exploratory factor analysis, and confirmatory factor analysis. The Mann-Whitney U test was used to examine gender differences across variables; partial correlation analysis, controlling for gender and grade, was employed to examine the intrinsic relationships among variables. Mplus 8.3 was used to construct the cross-lagged model, and maximum likelihood estimation with robust standard errors (MLR) was used for parameter estimation. Missing data arising from longitudinal attrition were coded as−99 in Mplus and handled using Full Information Maximum Likelihood (FIML) via the “MISSING ARE ALL (-99);” statement (38). FIML uses all available data to estimate model parameters without imputing missing values or deleting cases listwise, under the missing-at-random (MAR) assumption. Model fit was assessed using the Comparative Fit Index (CFI), Tucker-Lewis Index (TLI), Root Mean Square Error of Approximation (RMSEA), and Standardized Root Mean Square Residual (SRMR). For multi-group comparisons, the Satorra-Bentler scaled chi-square difference test was used to evaluate cross-group invariance.

## 3 Results

### 3.1 Common method bias test

To test for common method bias, Harman's single-factor test was first conducted by performing an unrotated exploratory factor analysis on all items (excluding demographic variables) across the three waves. The first factor explained 26.556% (T1), 26.033% (T2), and 30.815% (T3) of the variance, all below the 40% threshold. Subsequently, a more stringent single-factor confirmatory factor analysis (CFA) was performed in Mplus 8.3, in which all items were loaded onto a single latent factor. The model yielded a poor fit to the data at all three waves (T1: CFI = 0.590, TLI = 0.521, RMSEA = 0.092, SRMR = 0.076; T2: CFI = 0.599, TLI = 0.532, RMSEA = 0.097, SRMR = 0.081; T3: CFI = 0.626, TLI = 0.564, RMSEA = 0.101, SRMR = 0.079). It should be noted that Harman's single-factor test has limited sensitivity for detecting common method bias, and these tests alone cannot definitively rule out its presence. Nevertheless, the convergent evidence from both EFA and CFA, combined with the procedural controls implemented in this study (e.g., reverse-scored items, data collection across multiple time points), suggests that common method bias does not pose a serious threat to the validity of the findings.

### 3.2 Analysis of gender differences in adolescent physical exercise and aggressive behavior

Given that the Shapiro-Wilk test suggested that the assumption of normality was not met for both physical exercise and aggressive behavior scores across the three waves (*p* < 0.05), the nonparametric Mann-Whitney U test was used to examine gender differences (Table 1). Results showed significant gender differences in both physical exercise (*p* < 0.001) and aggressive behavior (*p* < 0.05). Specifically, boys' physical exercise scores across the three assessments (T1 = 27.870 ± 19.934, T2 = 29.780 ± 19.787, T3 = 29.240 ± 20.987) were all higher than those of girls (T1 = 16.050 ± 15.434, T2 = 15.910 ± 14.524, T3 = 16.690 ± 17.049).

**Table 1:** Mann-Whitney U test of gender.

Variable	PE1	PE2	PE3	AGG1	AGG2	AGG3
Mann-Whitney*U*	93,090.5	71,065	70,118	139,668	116,853.5	104,961
Wilcoxon*W*	241,875.5	199,336	179,864	291,193	245,124.5	224,277
Z	−10.724	−11.721	−10.307	−2.052	−2.404	−2.112
Asymptotic significance	< 0.001^***^	< 0.001^***^	< 0.001^***^	0.040^*^	0.016^*^	0.035^*^

PE, physical exercise; AGG, aggressive behavior; ^*^p < 0.05, ^**^p < 0.01, ^***^p < 0.001.

### 3.3 Cross-lagged analysis of adolescent physical exercise and aggressive behavior

A partial correlation analysis of adolescents' physical exercise and aggressive behavior was conducted after controlling for demographic variables (gender, educational stage) (Table 2). (1) There were significant positive correlations between T1 physical exercise, T2 physical exercise, and T3 physical exercise (*p* < 0.001), and there were also significant positive correlations between T1 aggressive behavior, T2 aggressive behavior, and T3 aggressive behavior (*p* < 0.001); (2) At the first assessment (T1), physical exercise was significantly negatively correlated with aggressive behavior (r = −0.183, *p* < 0.001); at the second assessment (T2), physical exercise was significantly negatively correlated with aggressive behavior (r = −0.189, *p* < 0.001); At the third measurement (T3), physical exercise was significantly negatively correlated with T3 aggressive behavior (r = −0.223, *p* < 0.001). These results indicate that physical exercise and aggressive behavior among adolescents exhibit stable and synchronous correlations across school years, making them suitable for cross-lagged analysis.

**Table 2:** Partial correlation analysis.

Control variables	Variable	PE1	PE2	PE3	AGG1	AGG2	AGG3
Gender & grade	PE1	1					
		PE2	0.523^***^	1				
		PE3	0.457^***^	0.510^***^	1			
		AGG1	−0.183^***^	−0.133^***^	−0.142^***^	1		
		AGG2	−0.191^***^	−0.189^***^	−0.144^***^	0.537^***^	1	
		AGG3	−0.167^***^	−0.190^***^	−0.223^***^	0.512^***^	0.585^***^	1

PE, physical exercise; AGG, aggressive behavior; ^*^p < 0.05, ^**^p < 0.01, ^***^p < 0.001.

**Figure 3:**
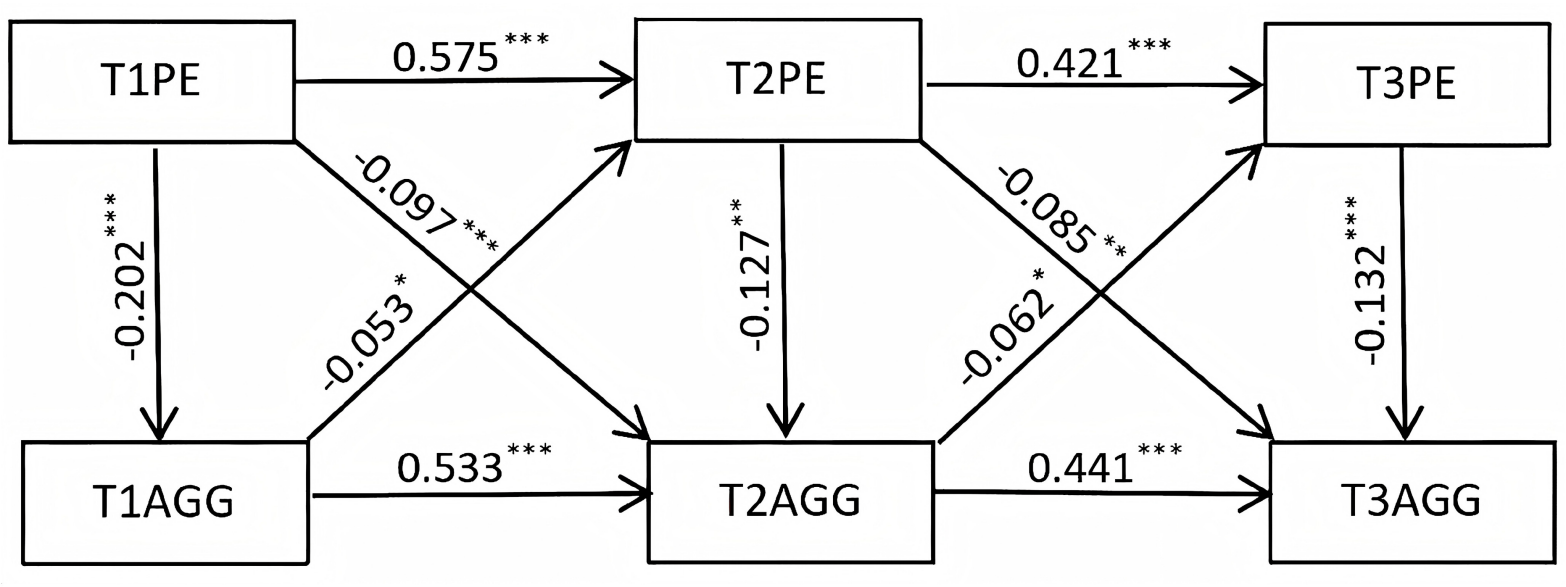
The cross-lagged effect model of physical exercise and aggressive behavior. PE, physical exercise; AGG, aggressive behavior. In accordance with recommendations for cross-lagged models (37), to control for systematic variance in the same variable across time points that is not fully explained by autoregressive paths, the model allows for correlation between the residuals of T1 and T3. *p < 0.05, **p < 0.01, ***p < 0.001.

A cross-lagged model was constructed using Mplus 8.3 software, and the model fit was assessed using the maximum likelihood method (Figure 2): χ^2^ = 7.414, χ^2^/df (2) = 3.707 (*p* = 0.025); Goodness-of-fit indices: CFI = 0.996, TLI = 0.975 (both > 0.90); Root Mean Square Error of Approximation (RMSEA) = 0.047 (< 0.06), 90% CI: 0.014, 0.086; Standardized Root Mean Square Residual (SRMR) = 0.016 (< 0.05). This indicates that the cross-lagged model of physical exercise and aggressive behavior exhibits acceptable model fit. The path coefficients of the cross-lagged model were used to examine the longitudinal association between physical exercise and aggressive behavior (Figure 3). (1) The effect of T1 physical exercise on T2 aggressive behavior was significant (β = −0.097, *p* < 0.001), and the effect of T2 physical exercise on T3 aggressive behavior was significant (β = −0.085, *p* < 0.01); (2) The effect of T1 aggressive behavior on T2 physical exercise was significant (β = −0.053, *p* = 0.038), and the effect of T2 aggressive behavior on T3 physical exercise was significant (β = −0.062, *p* = 0.019). The path coefficients of the model indicate that prior physical exercise was longitudinally associated with lower subsequent aggressive behavior, and prior aggressive behavior was longitudinally associated with lower subsequent physical exercise, supporting a bidirectional longitudinal association between the two variables.

To examine gender and grade-level differences in the cross-lagged model, unrestricted and restricted models were constructed separately with gender and educational stage as grouping variables (Table 3). Cross-group invariance was assessed using changes in fit indices and the Satorra-Bentler corrected chi-square test (39) to assess cross-group invariance. In the gender difference tests, both models fit well (χ^2^/df < 5, CFI ≥ 0.993, RMSEA ≤ 0.052); the Satorra-Bentler corrected chi-square test was not significant, with Δχ^2^_SB(8) = 11.94, *p* = 0.154; ΔCFI = −0.002 (|ΔCFI| < 0.01), ΔRMSEA = −0.014 (|ΔRMSEA| < 0.015), ΔSRMR = 0.012. In the test for differences across educational stages, both models also fit well (χ^2^/df < 5, CFI ≥ 0.994, RMSEA ≤ 0.039); The Satorra-Bentler adjusted chi-square test, Δχ^2^_SB(8) = 12.95, *p* = 0.113; ΔCFI = −0.004 (|ΔCFI| < 0.01), ΔRMSEA = −0.004 (|ΔRMSEA| < 0.015), ΔSRMR = 0.038. In the comparison between the two groups, the absolute values of ΔCFI were both less than 0.01 (40), and the absolute values of ΔRMSEA were both less than 0.015 (41). This indicates that imposing the cross-group equality constraint on path coefficients did not lead to a significant deterioration in model fit, and that the cross-lagged effects of adolescent physical exercise on aggressive behavior are stable across genders and school grades.

**Table 3:** Tests for gender and educational stage differences in cross-lagged effects.

Gender differences					Educational stage differences				
Model I metrics	Comparison of model metrics		Assumption: default correct model		Model I metrics	Comparison of model metrics		Assumption: default correct model	
	Unrestricted	Restricted	Metrics	Value		Restricted	Metrics	Metrics	Value
RMSEA	0.052	0.038	ΔRMSEA	−0.014	RMSEA	0.039	0.035	ΔRMSEA	−0.004
SRMR	0.019	0.031	ΔSRMR	0.012	SRMR	0.015	0.053	ΔSRMR	0.038
χ^2^/df	2.477	1.795	Δχ^2^/df	−0.682	χ^2^/df	1.837	1.689	Δχ^2^/df	−0.148
CFI	0.995	0.993	ΔCFI	−0.002	CFI	0.998	0.994	ΔCFI	−0.004
NNFI	0.968	0.983	ΔNNFI	0.015	NNFI	0.984	0.987	ΔNNFI	0.003
*P*	0.042	0.043	Δχ^2^_SB	0.154	Δχ^2^_SB	0.119	0.062	*P*	0.113

a. Satorra-Bentler corrected chi-square test for gender, Δχ^2^_SB (8) = 11.94, *p* = 0.154. b. Satorra-Bentler corrected chi-square test for educational stage, Δχ^2^_SB (8) = 12.95, *p* = 0.113. c. Δ RMSEA is the change in root mean square error of approximation, Δ SRMR is the change in standardized root mean square residual, Δ CFI is the change in comparative fit index, Δ NNFI is the change in normalized net fit index, and Δχ^2^/df is the change in chi-square-to-degrees-of-freedom ratio.

## 4 Discussion

### 4.1 Gender differences in adolescent physical exercise and aggressive behavior

The Mann-Whitney U test of data from the three assessments revealed stable, cross-temporal gender differences in adolescents' physical exercise (*p* < 0.001), with male adolescents scoring higher than female adolescents across all three waves, a finding consistent with the views of Zhu and Shu (42). This difference can be understood from both cognitive development and sociocultural construction perspectives. From a cognitive development perspective, individuals form gender identity through phased processes of cognitive development during childhood, and after acquiring gender constancy, they actively seek behavioral patterns consistent with their own gender (43); upon entering adolescence, males outperform females in terms of both exercise motivation and participation rates (44), as well as in terms of withdrawal from sports or individual engagement in sports during adolescence (45). From the sociocultural construction perspective, gender roles are the result of long-term sociocultural construction; individuals internalize society's differentiated expectations for the two sexes through observation, imitation, and reward-punishment mechanisms (46). In traditional Chinese gender norms, cultural expectations such as “men are breadwinners and women are homemakers” and “men should be strong and women gentle” are not innate but were shaped through the long-term construction of patriarchal social order (47), and continue to exert some influence on family socialization and social expectations. These cultural expectations may subtly influence adolescents' sports choices through pathways such as parenting styles, teacher expectations, and peer evaluation: boys are encouraged to participate in strength- and competitiveness-oriented activities such as football and basketball, whereas girls are directed toward flexibility- and cooperation-oriented activities such as gymnastics and volleyball (48). In summary, the dual effects of cognitive development and sociocultural factors jointly shape the stable, cross-temporal gender differences in adolescents' physical exercise.

The Mann-Whitney U test also revealed significant gender differences in adolescents‘ aggressive behavior across all three waves (*p* < 0.05), indicating that gender differences in aggressive behavior likewise exhibit cross-temporal stability. Social role theory (49) posits that gender-stereotyped behavior stems from society's differing role expectations for men and women. In traditional social division of labor, men are more often assigned to social roles involving competition, dominance, and protection, while women are more often assigned to nurturing, caregiving, and cooperation-oriented roles, thereby creating different behavioral expectations and norms. Building on this, society holds differentiated tolerance toward aggressive behavior across genders: male aggression tends to be perceived as a sign of “courage” or “responsibility” and is to some extent tacitly accepted or even encouraged, whereas female aggression tends to be viewed as “unladylike” and faces stronger social sanctioning pressure (50). At the same time, elevated androgen levels during adolescence directly promote aggressive impulsivity in males (51), and combined with the internalization of social role expectations, this leads males to adopt more direct, overt aggressive behavior when facing frustrating situations, while females are more likely to inhibit aggressive impulses or express dissatisfaction through indirect, relational means (50). In summary, the cross-temporal stability of gender differences in adolescents' aggressive behavior may be the result of multiple forces—including biological factors, social role expectations, and differentiated social tolerance—working in concert.

### 4.2 The bidirectional longitudinal association between physical exercise and aggressive behavior in adolescents

The intensity, duration, and frequency of physical exercise are not merely external quantitative indicators of activity but may also reflect an individual's underlying psychological state and internal capabilities, constituting an externalized behavioral manifestation. Based on this premise, the study quantified adolescents' physical exercise characteristics using indicators such as exercise frequency, duration, and intensity. Through partial correlation analyses controlling for gender and grade, and after confirming the cross-temporal, synchronous correlation between adolescents' physical exercise and aggressive behavior, the study utilized data from three assessment waves to construct a cross-lagged model. The results supported a bidirectional longitudinal association: prior physical exercise was longitudinally associated with lower subsequent aggressive behavior, and prior aggressive behavior was longitudinally associated with lower subsequent physical exercise.

Berkowitz's revised frustration-aggression hypothesis posits that aggression is caused by frustration resulting from goal obstruction. This frustration creates a state of readiness for aggression; whether it ultimately manifests as aggressive behavior depends on situational cues and individual inhibitory factors (52). Physical exercise may provide a short-term outlet, offering adolescents a socially acceptable channel for the expression of aggressive behavior and allowing them to safely release negative emotions accumulated from daily frustrations, thereby interrupting the chain of transformation from frustration to aggressive behavior. The General Aggression Model (GAM) posits that aggressive behavior is not directly caused by a single factor (such as frustration) but rather results from the interaction between personal and situational factors, which ultimately influence the individual's current internal state (cognitive, emotional, and physiological arousal) (53). Based on this, it is plausible to speculate that long-term physical exercise may continuously optimize individuals' cognitive, emotional, and arousal pathways by enhancing executive function and reducing negative emotions, thereby potentially inhibiting the occurrence of aggressive behavior. However, this speculation awaits direct testing in future research. Existing empirical research indicates that reducing and preventing aggressive behavior in children and adolescents is crucial for their future growth and development, mental health, and social adaptation; if aggressive behavior occurs, it may trigger a series of problematic behaviors, such as peer rejection, academic difficulties, and internalizing problems (20). Meanwhile, meta-analytic evidence indicates that regular physical exercise can significantly reduce the overall level of aggressive behavior in children and adolescents (13). It can also be speculated that physical exercise may help adolescents develop self-control and emotion regulation skills during participation (2, 4), thereby reducing aggressive behavior and externalizing problem behaviors. However, this speculated pathway awaits direct testing in future research by incorporating mediating variables. In summary, the present study suggests that encouraging adolescents to actively engage in beneficial physical exercise may be associated with reduced aggressive behavior.

The study also found that aggressive behavior was negatively associated with subsequent physical exercise; that is, adolescents with higher levels of aggressive behavior engaged in less physical exercise later on. The frustration-aggression hypothesis suggests that if aggressive behavior successfully helps an individual achieve a goal, that behavior will be reinforced, thereby reducing interest in other positive alternative behaviors (54). In other words, adolescents with high levels of aggression may have learned that “aggression is an effective problem-solving strategy,” and thus no longer feel the need to engage in physical exercise to vent the negative effects of frustration. When aggressive behavior satisfies an individual's need for a sense of control, status acquisition, or emotional regulation, the appeal of physical exercise diminishes relatively. The General Aggression Model (53) posits that following an aggressive act, the individual enters a state of self-depletion. Once aggressive patterns become entrenched, the individual becomes trapped in an “aggression cycle,” with cognitive structures, emotional states, and arousal levels deviating from adaptive states. Based on this, it is plausible to speculate that aggressive individuals may therefore find it difficult to proactively engage in physical exercise—an activity requiring self-discipline and persistence. This speculation awaits testing in future research. At the same time, the act of aggression is often accompanied by negative emotions (shame, guilt) and negative conditioned responses to the activity context. This dual emotional and cognitive depletion leads aggressive individuals to prefer sedentary, low-energy activities over physical exercise. In group sports settings, peers are more likely to refuse to team up with or participate in activities alongside highly aggressive individuals, resulting in a significant reduction in their opportunities to engage in physical exercise (24). Based on this, and in conjunction with the results of data analysis, the present study suggests that intervening to reduce aggressive behavior may be associated with greater engagement in physical exercise.

It is worth noting that the cross-lagged path coefficients identified in this study (β = −0.053 to −0.097), while statistically significant, are relatively modest in magnitude. This is not uncommon in longitudinal research. After controlling for the autoregressive effects of each variable, cross-lagged paths represent “net effects” that are typically attenuated by strong autoregressive stability (55). In other words, the stronger the temporal stability of the variables themselves, the more limited the additional variance that cross-lagged paths can explain. Therefore, the value of the present findings lies not primarily in the magnitude of the effects, but in demonstrating the existence and stability of bidirectional longitudinal associations between physical exercise and aggressive behavior across gender and educational stages. From a practical standpoint, even modest individual path coefficients should not be overlooked when aggregated across adolescent populations. Long-term, stable micro-effects may accumulate over time and carry meaningful implications.

Finally, the present study found a bidirectional longitudinal association between physical exercise and aggressive behavior, and the cross-lagged effects were consistent across gender and educational stages. This finding is consistent with previous research (4) and supports the applicability of this effect across gender and educational stages. This suggests that intervention programs should not be limited to a unidirectional approach focused solely on promoting health through exercise, as adolescents with high levels of aggression may face a vicious cycle in which the more aggressive they become, the more likely they are to withdraw from physical activity, thereby missing opportunities for improvement. Therefore, intervention strategies could consider bidirectional and tiered approaches, not only using physical activity to engage and prevent aggressive behavior, but also developing specialized programs to proactively identify and re-engage adolescents who have disengaged from physical exercise due to aggressive tendencies. These findings raise the possibility that physical education programs could benefit from cultivating sportsmanship and social interaction skills while strengthening emotional self-management and control abilities. In addition, as one potential direction for future intervention research, non-competitive roles (such as equipment management, tactical record-keeping, or assistant refereeing) could be explored as a strategy to first reduce the sense of threat regarding participation among identified high-aggression students, before gradually transitioning them into cooperative sports. It should be emphasized that this specific suggestion is speculative and its effectiveness awaits direct empirical testing in future studies.

## 5 Limitations and future directions

Several limitations of this study should be acknowledged. First, the sample was drawn exclusively from eight secondary schools in Jiangxi Province, which limits the geographical representativeness of the findings. Cultural traditions, educational competition, and the availability of school sports resources in Jiangxi may differ from those in other regions, all of which may influence adolescents' physical activity participation and the expression of aggressive behavior. Caution is therefore warranted when generalizing these findings to the broader population of Chinese adolescents or to other cultural contexts. Future research should extend the sample to multiple provinces and cities to test the cross-regional stability of the conclusions.

Second, the exclusive reliance on self-report questionnaires may have introduced recall bias and social desirability bias. Although both exploratory and confirmatory factor analyses suggested that common method bias was not a serious concern, the use of a single data source precludes fully ruling out its potential influence. In addition, the internal consistency values of both the Physical Activity Rating Scale (PARS-3; Cronbach's α = 0.478–0.594) and the Buss-Perry Aggression Questionnaire (BPAQ; Cronbach's α = 0.645–0.698) were below conventional thresholds in the current sample. It should be noted that the PARS-3 is a composite index derived from a multiplicative formula combining intensity, frequency, and duration, which capture distinct aspects of physical activity rather than a single homogeneous construct; Cronbach's alpha is therefore not the most appropriate indicator of its reliability, and the test-retest reliability assessed using the intraclass correlation coefficient was acceptable to good (ICC = 0.738–0.732). Nevertheless, the relatively low internal consistency may still have produced an attenuation effect, rendering the estimates of the associations more conservative and potentially underestimating the true effect sizes. The significant results obtained under these conservative conditions further strengthen confidence in the robustness of the core findings. Furthermore, Item 11 of the BPAQ was reassigned from the Verbal Aggression dimension to the Anger dimension based on statistical and theoretical considerations. Although this adjustment is justifiable, it may limit the comparability of the present findings with studies that employed the original factor structure, and it also suggests that the applicability of this scale in contemporary adolescent populations warrants further examination. Future research should adopt multi-method and multi-informant approaches—such as objective physical activity monitoring, teacher evaluations, and parent reports—or employ measurement tools that are more aligned with the contemporary context.

Third, this study employed the traditional cross-lagged panel model (CLPM), which conflates between-person differences with within-person variation, making it difficult to distinguish stable trait-like differences from dynamic within-person changes. Recent methodological advances have proposed the Random Intercept Cross-Lagged Panel Model (RI-CLPM), which separates between-person differences from within-person variation and thereby provides cleaner estimates of within-person dynamics (56). Future research could consider applying RI-CLPM to further validate whether the bidirectional associations observed here also hold at the within-person level.

Finally, this study examined only the direct bidirectional pathways between physical exercise and aggressive behavior. The mechanisms discussed above—such as executive function, emotion regulation, and self-depletion—are speculative and await direct testing in future research through the incorporation of corresponding mediating variables. In summary, future research should focus on expanding the sample to multiple regions, adopting multi-method and multi-informant measurement approaches, applying more refined statistical methods such as RI-CLPM, and conducting in-depth investigations of mediating mechanisms, so as to provide a more robust theoretical basis for the development of targeted physical activity intervention programs.

## 6 Conclusions

Both physical exercise and aggressive behavior among adolescents exhibit stable gender differences; physical exercise and aggressive behavior are linked by a bidirectional longitudinal association rather than a unidirectional one. This implies that higher levels of physical exercise are associated with lower levels of aggressive behavior, and addressing aggressive tendencies is associated with greater engagement in physical exercise. The cross-lagged effects between physical exercise and aggressive behavior demonstrate stability across genders and educational stages.

## Data Availability

The raw data supporting the conclusions of this article will be made available by the authors, without undue reservation.
